# Pyroptosis-related lncRNAs: A novel prognosis signature of colorectal cancer

**DOI:** 10.3389/fonc.2022.983895

**Published:** 2022-11-30

**Authors:** Xing Cai, Xiaoqing Liang, Kun Wang, Yin Liu, Mengdi Hao, Huimin Li, Xiaofang Dai, Lei Ding

**Affiliations:** ^1^ Cancer Center, Union Hospital, Tongji Medical College, Huazhong University of Science and Technology, Wuhan, China; ^2^ Department of Oncology, Beijing Shijitan Hospital, Capital Medical University, Beijing, China

**Keywords:** pyroptosis, long non coding RNA, colorectal cancer, prognosis, TCGA

## Abstract

Pyroptosis is a newly discovered programmed cell death mechanism involved in tumorigenesis. Long non-coding RNAs (lncRNAs) have been implicated in colorectal cancer (CRC). However, the potential role of pyroptosis-related lncRNAs (PRLs) in CRC remains unelucidated. Therefore, we retrieved transcriptomic data of CRC patients from The Cancer Genome Atlas (TCGA). With the use of univariate and multivariate Cox proportional hazards regression models and the random forest algorithm, a new risk model was constructed based on eight PRLs: *Z99289.2, FENDRR, CCDC144NL-ASL, TEX41, MNX1-AS1, NKILA, LINC02798*, and *LINC02381*. Then, according to the Kaplan–Meier plots, the relationship of PRLs with the survival of CRC patients was explored and validated with our risk model in external datasets (Gene Expression Omnibus (GEO) databases; GEO17536, n = 177, and GSE161158, n = 250). To improve its clinical utility, a nomogram combining PRLs that could predict the clinical outcome of CRC patients was established. A full-spectrum immune landscape of CRC patients mediated by PRLs could be described. The PRLs were stratified into two molecular subtypes involved in immune modulators, immune infiltration of tumor immune microenvironment, and inflammatory pathways. Afterward, Tumor Immune Dysfunction and Exclusion (TIDE) and microsatellite instability (MSI) scores were analyzed. Three independent methods were applied to predict PRL-related sensitivity to chemotherapeutic drugs. Our comprehensive analysis of PRLs in CRC patients demonstrates a potential role of PRLs in predicting response to treatment and prognosis of CRC patients, which may provide a better understanding of molecular mechanisms underlying CRC pathogenesis and facilitate the development of effective immunotherapy.

## Introduction

Colorectal cancer (CRC) is the third most common cancer and the second leading cause of cancer death worldwide (1). In 2018, there were about 1.8 million new cases and 880,000 deaths of CRC globally (2). CRC is a group of heterogeneous diseases; therefore, the genetic makeup, age, family history, ethnicity, and lifestyle of patients varied greatly (3). Interestingly, pyroptosis-related genes and long non-coding RNAs (lncRNAs) may play a vital role as independent molecular biomarkers of tumor diagnosis and prognosis (4). Considering the high mortality rate, a new series of biomarkers suitable for CRC prognosis shall be urgently needed.

Pyroptosis, which belongs to inflammatory cell death, is distinct from apoptosis and ferroptosis (5). It is characterized by cell swelling, pore formation, osmotic lysis, Gasdermin family-mediated pore-forming, cell lysis, and release of inflammatory factors, including interleukin IL-18, IL-1β, and high mobility group box 1 (HMGB1) (6–8). The Gasdermin superfamily is the executioner of pyroptosis (9). Downregulation of GasderminD (GSDMD) in CRC was associated with poor prognosis in CRC, suggesting that the Gasdermin family may be a potential therapeutic target for CRC (10).

LncRNA is a non-coding RNA with transcripts longer than 200 nucleotides (11). Accumulating evidence suggests that lncRNAs may participate in various human diseases and disorders (12). The lncRNAs were associated with metastasis and prognosis of CRC. For example, *HOTAIR* was associated with poor prognosis in CRC (13). H19 promotes CRC metastasis *via* binding to *hnRNPA2B1* (14). Metastasis-Associated Lung-Adenocarcinoma Transcript 1 (MALAT1) was associated with metastasis and survival of CRC patients (15). The high expression level of *RAMS11* in primary CRC tumors may predict a worse outcome (16). Although lncRNAs were involved in the progression and prognosis of CRC, relevant evaluation of gene signature based on lncRNAs in CRC has yet to be explored.

In this study, we obtained colon adenocarcinoma (COAD) and rectal adenocarcinoma (READ) RNA-seq and clinical data from The Cancer Genome Atlas (TCGA) and explored the prognostic significance of PRLs using bioinformatic and statistical analyses. Eight PRLs holding prognostic values in COAD and READ patients were used to construct a PRL model. In addition, we developed a nomogram to assess prognosis. Its predictive power was verified using the Gene Expression Omnibus (GEO) databases. Based on the median risk scores, CRC patients in TCGA were divided into high- and low-risk subtypes. Then, prognosis, the functional pathways consisting of differential expression genes, immune cell infiltration, microsatellite instability (MSI) status, tumor mutational burden (TMB), and chemotherapeutic drug sensitivity were evaluated.

## Materials and methods

### Data resources and preparation

**Figure 1:**
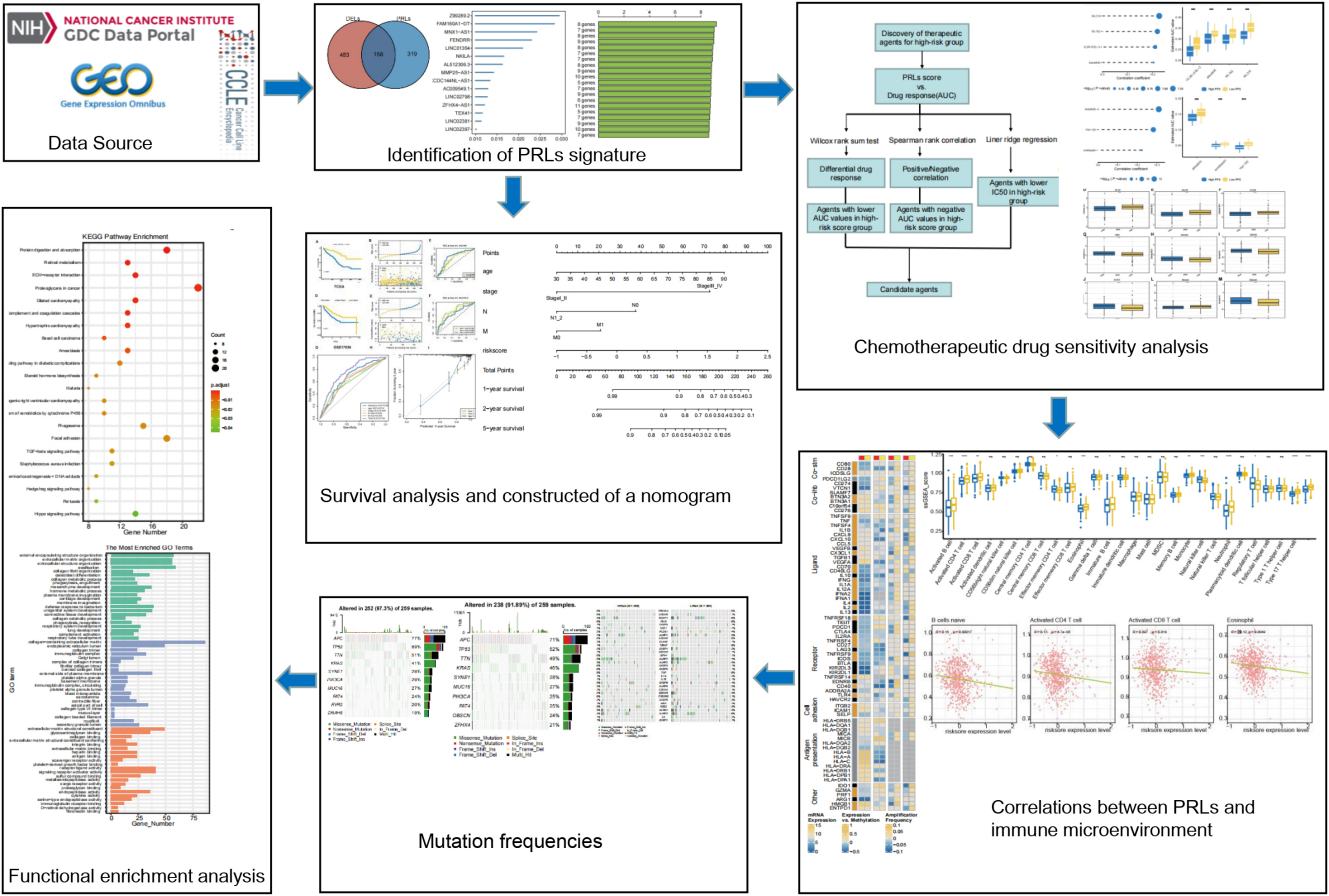
Flowchart of the whole study. PRLs, pyroptosis-related long non-coding RNAs. * represents p < 0.05, ** represents p < 0.01, *** represents p < 0.001, ns represents no significant.

The DNA methylation, gene expression profile, and corresponding clinical information data of CRC patients were downloaded from TCGA database (https://cancergenome.nih.gov/). After screening, we excluded samples with insufficient clinical information. A total of 571 CRC cases containing mRNA expression and corresponding clinical data were used as a training cohort. The fragments per kilobase of transcript per million mapped reads (FPKM) data of TCGA cohort were then converted into transcripts per kilobase million (TPM) data for the next analysis. Then, two independent datasets with expression data and detailed clinical information were downloaded from the GEO datasets (GSE17536, n = 177; GSE161158, n = 250; https://www.ncbi.nlm.nih.gov/geo/) and were used as validation sets. Gene Ensemble ID was annotated as Gene Symbol through GENE CODE v27. After that, expression profile and somatic mutations data of human cancer cell lines were obtained from the Broad Institute Cancer Cell Line Encyclopedia (CCLE) project (https://portals.broadinstitute.org/ccle/) (17). A total of 33 pyroptosis-related genes (PRGs) were obtained from previous literature (**Supplementary Table S1**) (18–22). The workflow of this study is described in **Figure 1**.

### The construction and validation of pyroptosis-related lncRNA prognostic model

The R package “*DESeq2*” was used to identify differentially expressed lncRNAs (DELs) between normal and tumor samples in TCGA cohort (**Supplementary Table S2**). Spearman’s correlation analysis between lncRNAs and PRGs was set to identify PRLs and acquisition of 477 PRLs (|R^2^| > 0.4 and p< 0.05, **Supplementary Table S3**). Take the intersection of 477 PRLs and 641 DELs (padj.< 0.05 and |log2FC| > 1), and 158 PRLs are obtained. Then, a univariate Cox proportional risk regression model was used to select candidate survival-related PRLs, and p< 0.01 was set as the threshold (**Supplementary Table S4**). Then, the random survival forests-variable hunting (RSFVH) algorithm was used to further filter the genes. The best log-rank p-values of the Kaplan–Meier (KM) analysis were used to establish the final PRL gene signature. A combination of eight PRLs was chosen for establishing the risk gene model because it had a relatively significant −log_10_ p-value and the smallest number of genes. Finally, according to the eight lncRNAs obtained by the RSFVH, we performed a multivariate regression analysis to construct the prognostic model.


$$\begin{array}{l} \text{Risk score}=0.12095\;*\;expZ99289.2\;+\;0.20536\;*\;expMNX1-AS\;+\;(\;-0.21082831\;*\;\\ \text{exp}FENDRR\text{) }+\;0.10058\;*\;expCCDC144NL-AS1\;+\;0.04314\;*\;LINC02798\;+\;\\ 0.053611\;*\;expTEX41\;+\;0.08983\;*\;expLINC02381 \end{array}$$

According to the median risk scores, patients in the training cohort were divided into low-risk and high-risk groups. Then, the Kaplan–Meier (K-M) plots were used to compare the overall survival (OS) time of the high- and low-risk groups. Before the construction of the nomogram, Schoenfeld’s residuals test was used to test the proportional hazards assumption in the Cox model. Finally, with the use of risk scores, age, and N and M, a nomogram was constructed. Time-dependent receiver operating characteristic (ROC) curves were used to evaluate the performance of this model for predicting prognosis. In addition, to depict the predictive value, corresponding calibration plots of the nomogram were used for 1-, 2-, and 5-year survival events. In the validation set, the risk scores were calculated using the same formula as that in the training set. All patients in the validation set were subdivided into low-risk and high-risk groups. Then, K-M plots were used to compare the OS between the two groups.

### Gene set enrichment analysis

The gene set enrichment analysis (GSEA) was performed by using the R package “Clusterprofiler” to compare related pathways and biological processes between the high- and low-risk groups. The hallmark gene set contains 50 representative pathways, which cover clearly defined sets of genes involved in development and immunity, which were analyzed in this study. Gene sets with nominal (NOM) p< 0.05 and |NES| > 1 were considered significant based on the user guide of GSEA (23).

### Chemotherapeutic response analysis

Drug sensitivity was calculated using data from three databases. The drug sensitivity data of human cancer cell lines (CCLs) were downloaded from the Cancer Therapeutics Response Portal (CTRP; https://portals.broadinstitute.org/ctrp) and PRISM Repurposing dataset (PRISM, https://depmap.org/portal/prism/). Both CTRP and PRISM datasets contain sensitivity to compounds in CCLS and provide the area under the dose–response curve (area under the curve (AUC)) values as a measure of drug sensitivity. The lower the value of AUC, the higher the sensitivity of the treatment (17). With the use of the ridge regression algorithm provided by the R package “pRRophetic”, the chemotherapeutic response of each patient was determined by the half-maximal inhibitory concentration (IC50) based on a pharmacogenomics database called Genomics of Drug Sensitivity in Cancer (GDSC) (https://www.cancerrxgene.org/) (24, 25).

### Immune cell infiltration analysis

To explore interactions of PRLs with tumor immune microenvironment (TME) in CRC, the currently acknowledged seven methods to calculate the immune infiltration status including ssGSEA, CIBERSORT, Estimate, MCP_counter, Quanti-seq, TIMER, and xCELL were used to analyze the infiltration level between the high- and low-risk groups of our constructed model (26, 27). The R package “matfools” was used to calculate TMB, while package “PreMSIm” was used to predict the MSI status. Finally, Tumor Immune Dysfunction and Exclusion (TIDE; http://tide.dfci.harvard.edu/) was used to evaluate the possibility of tumor immune escape in the gene expression profile between the low- and high-risk groups (28).

### Statistical analysis

Statistical analysis was performed with the R software 4.1.2. Spearman’s correlation analysis was used to identify PRLs. U tests were used when appropriate. If not stated above, p< 0.05 is considered significant.

## Results

### Identification of pyroptosis-related lncRNA signature

**Figure 2:**
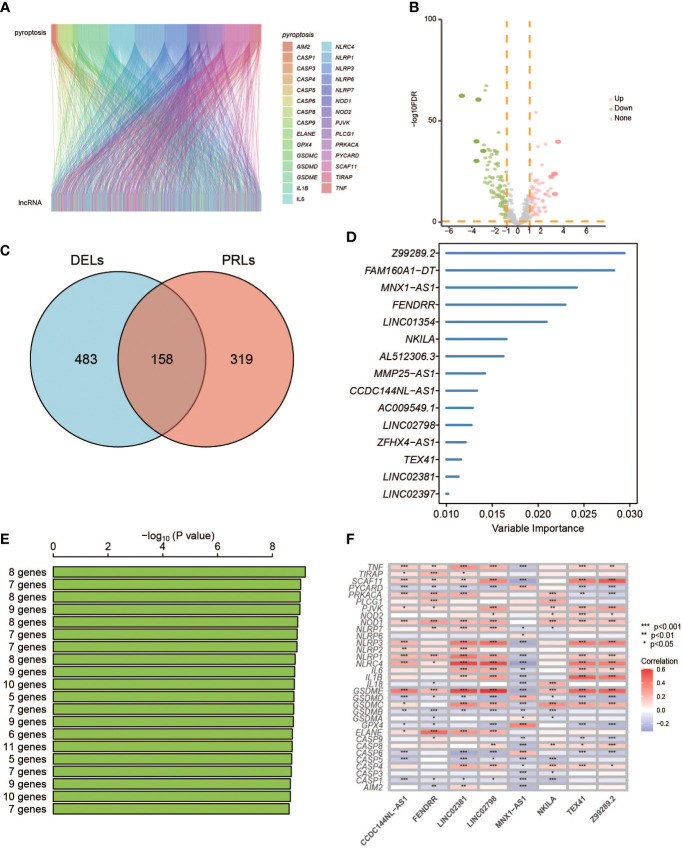
Identification of PRLs and development of a PRL signature. **(A)** Sankey diagrams to describe the associations between PRGs and lncRNAs. **(B)** Volcano diagrams exhibit differentially expressed lncRNAs. **(C)** The Venn diagrams identify the intersects of DELs and PRLs. **(D)** Random survival forest analysis on the screened 15 lncRNAs. **(E)** The top eight lncRNAs were sorted according to the p-values of K-M plots. **(F)** Heatmap shows the correlation of 33 PRGs with eight lncRNAs. PRGs, pyroptosis-related genes; lncRNAs, long non-coding RNAs; DELs, differentially expressed lncRNAs; KM, Kaplan–Meier. * represents p < 0.05, ** represents p < 0.01, *** represents p < 0.001.

**Figure 3:**
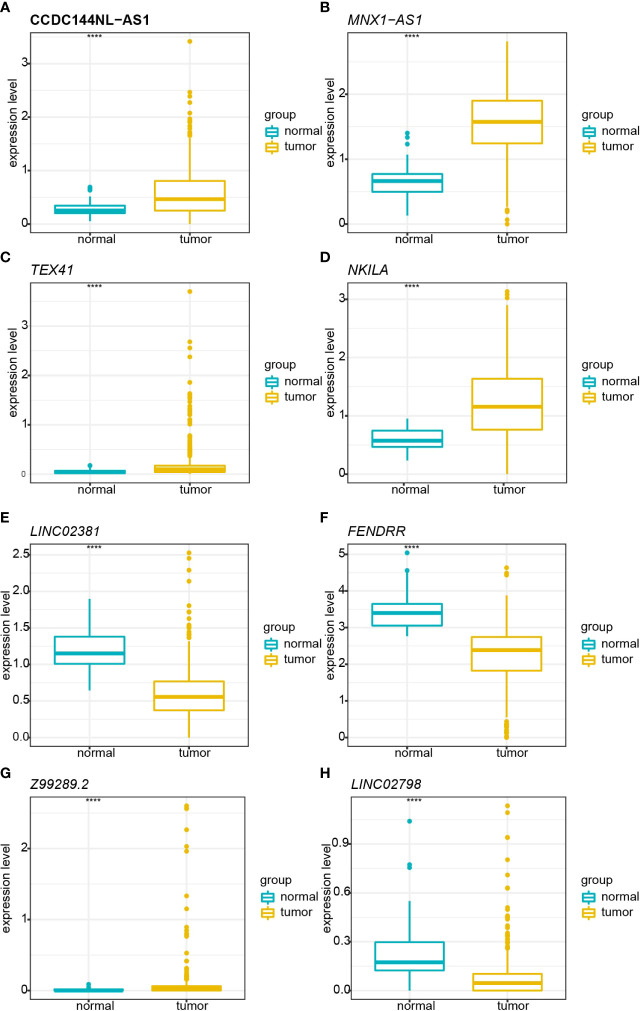
Boxplot shows the comparisons of relative expression in normal and tumor tissue in TCGA cohort. **(A)** Comparison of the relative expression of *CCDC144NL-AS1* in normal and tumor tissues. **(B)** Comparison of the relative expression of *MNX1-AS1* in normal and tumor tissues. **(C)** Comparison of the relative expression of *TEX41* in normal and tumor tissues. **(D)** Comparison of the relative expression of *NKILA* in normal and tumor tissues. **(E)** Comparison of the relative expression of *LINC02381* in normal and tumor tissues. **(F)** Comparison of the relative expression of *FENDRR* in normal and tumor tissues. **(G)** Comparison of the relative expression of *Z99289.2* in normal and tumor tissues. **(H)** Comparison of the relative expression of *LINC02798* in normal and tumor tissues. TCGA, The Cancer Genome Atlas. **** represents p < 0.001.

**Figure 4:**
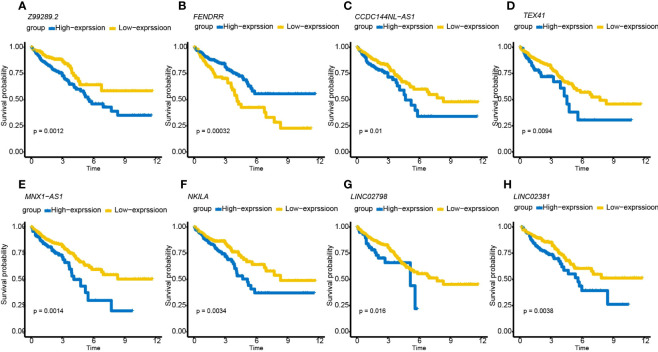
K-M survival curves showing that CRC patients with different expression levels of the eight PRLs had different survival probabilities. **(A–H)** K-M plots of lncRNAs *Z99289.2, FENDRR, CCDC144NL-AS1, MNX1-AS1, NKILA, LINC02798*, and *LINC02381*. K-M, Kaplan–Meier; CRC, colorectal cancer; PRLs, pyroptosis-related lncRNAs.

A total of 998 patients with CRC gene expression data and relative clinical information were included in this study. The clinical information of TCGA cohort is summarized in **Supplementary Table S5**. We obtained 33 pyroptosis-related genes from the literature and analyzed their associations with lncRNAs in CRC patients to identify PRLs (Spearman’s threshold value |R^2^| > 0.4 and p< 0.05, **Figure 2A**). Next, differently expressed lncRNAs (DELs) in CRC patients between normal and cancer tissues were obtained. Taking genes shared by DELs and PRLs, we obtained 158 PRLs to do the next univariate Cox proportional hazards analysis and identify PRLs that are significantly related to OS of CRC patients (p< 0.05, **Figures 2B, C**). Subsequently, 15 lncRNAs (*ALMS1-IT1, AC004846.1, LINC01354, SNHG7, Z99289.2, LINC02397, AC009549.1, GAS1RR, AL354836.1, LINC02381, AL137026.1, LINC00702, AC012313.5, ELFN1-AS1*, and *LINC02798*) were screened out with random forest supervised classification algorithm (**Figure 2D**). The K-M analysis was performed to screen genes that would be best for a risk model. The final prognostic signature is composed of eight PRLs (*TEX41, Z99289.2, LINC02798, CCDC144NL-AS1, LINC02381, MNX1-AS1, FENDRR*, and *NKILA*) based on a bigger −log_10_ p_log-rank_ value and a smaller number of genes (**Figure 2E**). The high correlation between eight PRLs and pyroptosis-related genes is shown in **Figure 2F**. The expression level of *CCDC144NL-AS1, MNX1-AS1, TEX41, NKILA*, and *Z99289.2* was higher in tumor tissue. In the meantime, the expression of *LINC02381* and *LINC02798* in the tumor is lower than that in normal tissue in CRC patients (**Figures 3A–H**). Furthermore, the expression levels of *lncRNAs TEX41, Z99289.2, LINC02798, CCDC144NL-AS1, LINC02381, MNX1-AS1*, and *NKILA* positively correlated with the OS. A higher expression level of lncRNA *FENDRR* correlated with better survival probability in TCGA CRC cohort (**Figures 4A–H**).

### Survival prediction performance of the model

**Figure 5:**
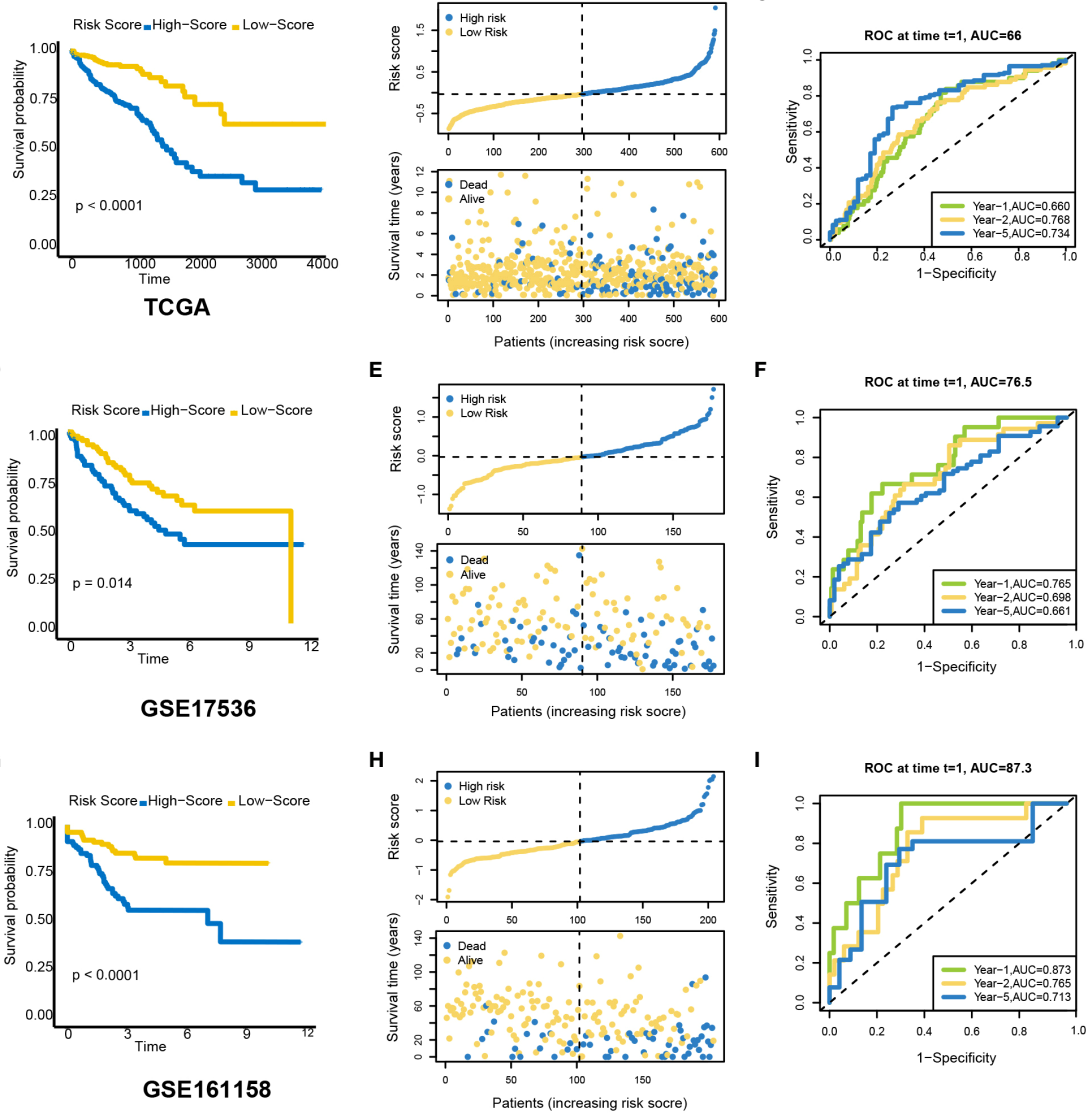
Prognostic value of a candidate risk model including eight PRLs. TCGA: **(A)** K-M plots for OS in the high-risk and low-risk groups stratified by PRLs. **(B)** Distribution of risk scores and survival status in TCGA cohort. **(C)** ROC curves for the eight lncRNAs. GSE17536: **(D)** K-M curves for OS in the high-risk and low-risk groups stratified by PRLs. **(E)** Distribution of risk scores and survival status in the validation cohort. **(F)** ROC curves for the eight lncRNAs. GSE161158: **(G)** K-M curves for OS in the high-risk and low-risk groups stratified by PRLs. **(H)** Distribution of risk scores and survival status in the validation cohort. **(I)** ROC curves for the eight lncRNAs. PRLs, pyroptosis-related lncRNAs; TCGA, The Cancer Genome Atlas; K-M, Kaplan–Meier; OS, overall survival; ROC, receiver operating characteristic.

CRC patients were divided into low- and high-risk groups according to the median risk scores. Based on the Kaplan–Meier survival curves, the low-risk group had a higher survival probability in TCGA dataset (**Figure 5A**). The risk scores and survival status were distributed in scatter plots. Patients with a higher risk score had shorter OS and a higher mortality rate (**Figure 5B**). According to the ROC curves, PRLs might predict OS in TCGA cohort, with an AUC at 1, 2, and 5 years of 0.660, 0.768, and 0.734, respectively (**Figure 5C**). Next, the GEO was used as an external dataset to validate the prognostic ability of PRLs. The GSE17536 and GSE161158 were explored as the validation cohorts. The risk scores in the validation cohorts were calculated using the same formula as in TCGA cohort. The results were consistent with the previous conclusion. Patients in the validation cohorts with a higher risk score had a lower survival rate compared to those with a low-risk score (**Figures 5D–I**).

### Stratified analysis and nomogram constructed based on the pyroptosis-related lncRNAs

**Figure 6:**
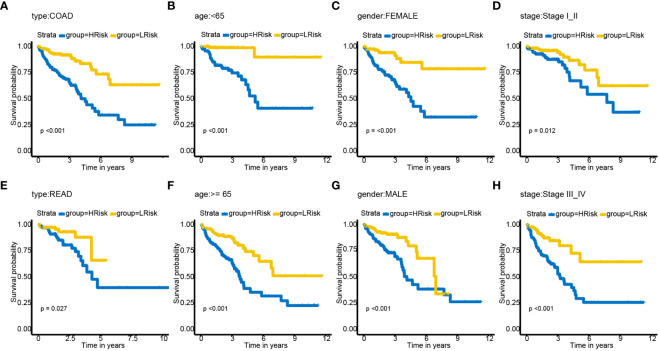
Subgroup analysis of OS for CRC patients based on PRLs. **(A)** cancer type—COAD. **(B)** Age< 65. **(C)** Gender—female. **(D)** Tumor stage I_II. **(E)** Type—READ. **(F)** Age ≥ 65. **(G)** Gender—male. **(H)** Tumor stage III_IV. COAD, colon adenocarcinoma; READ, rectal adenocarcinoma; OS, overall survival; CRC, colorectal cancer; PRLs, pyroptosis-related lncRNAs.

**Figure 7:**
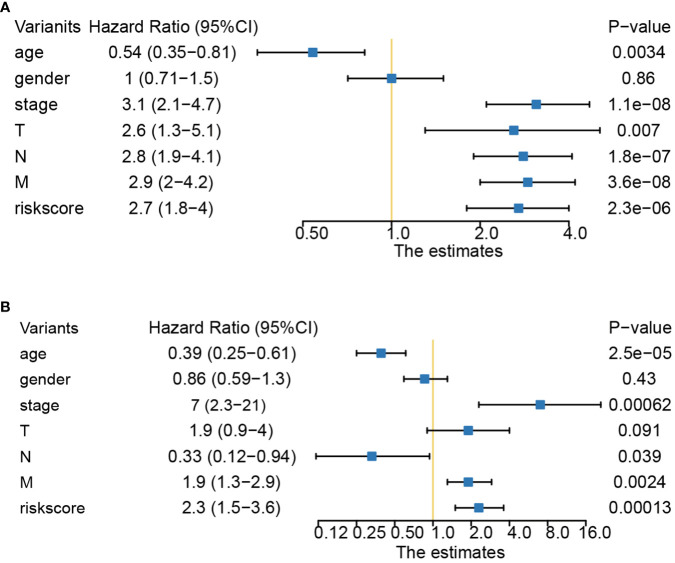
Univariate and multivariate Cox analyses of clinical factors and risk score with OS. **(A)** Risk score was an independent predictor as demonstrated by univariate analyses. **(B)** Risk score was an independent predictor as demonstrated by multivariate analyses. OS, overall survival.

**Figure 8:**
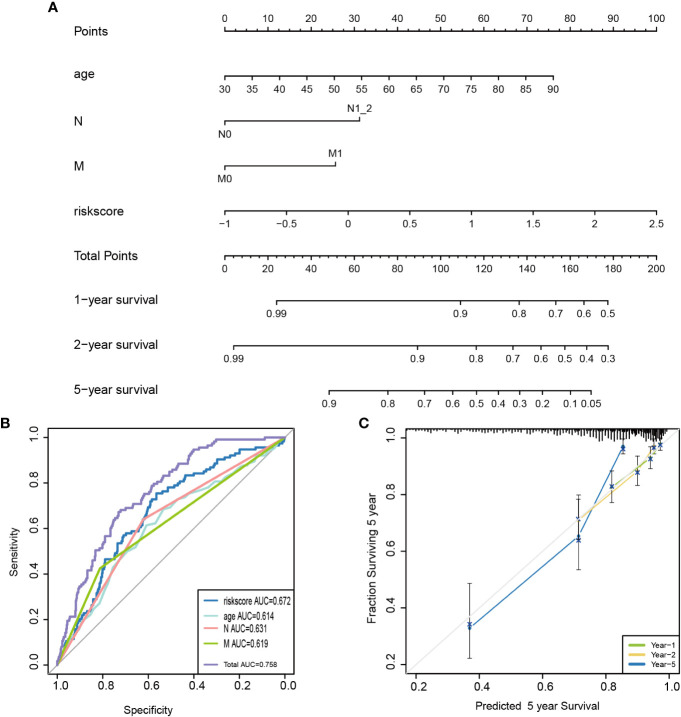
Construction of a nomogram for survival prediction. **(A)** A candidate nomogram was developed to predict 1-, 2-, and 5-year survival rates. **(B)** A comparison of 5-year ROC curves with common clinical characteristics (age and N and M status) and risk score, indicating the superiority of this new risk score. **(C)** Calibration curves described nomogram prediction abilities for 1-, 2-, and 5-year survival rates. ROC, receiver operating characteristic.

The relationships between risk scores and different clinicopathological factors (age, gender, tumor type, and clinical stage) were investigated. The patients with a low-risk score had a better survival probability than those with a high-risk score (**Figures 6A–H**). Then, prognostic factors of CRC patients were identified independently. The univariate and multivariate Cox analyses were performed to screen factors with prognostic power (**Figures 7A, B**). Schoenfeld’s test was used to examine the quality of factors used to build a nomogram (**Supplementary Figure S1**). Then, a nomogram was constructed to calculate the 1-, 2-, and 5-year survival probability of a CRC patient based on tumor grade, age, and risk score (**Figure 8A**). The AUC experiments on the nomogram model showed higher accuracy for OS at 1, 2, and 5 years in TCGA cohort (**Figure 8B**). In addition, based on calibration plots, the predicted power was close to an ideal curve (**Figure 8C**), which demonstrated that this nomogram had a good prognostic performance in CRC and was helpful to improve the clinical utility of PRL risk score.

### Pyroptosis-related lncRNA-associated different functional pathways and somatic mutation landscape

**Figure 9:**
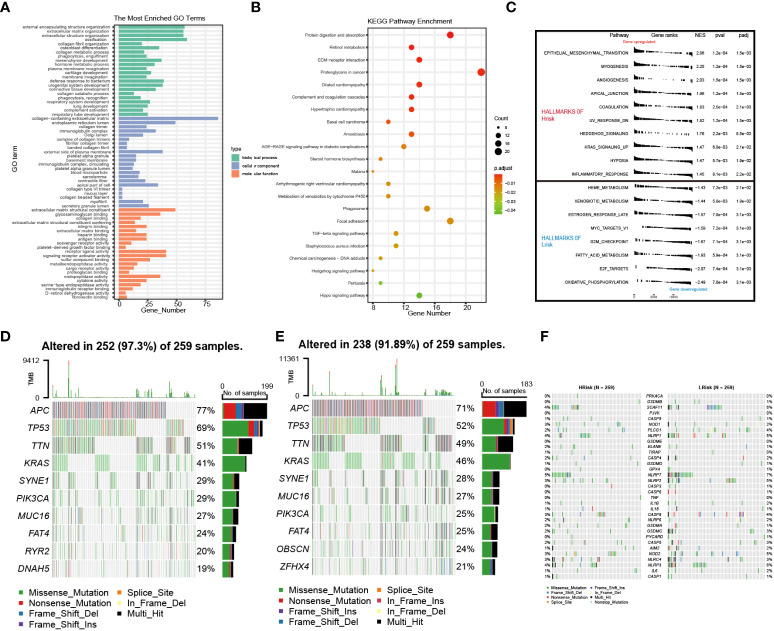
Enrichment analysis and cell mutation landscape. **(A, B)** GO and KEGG enrichment analyses. **(C)** GSEA functional enrichment analysis. **(D, E)** The waterfall plot of 10 mutated genes in high- and low-risk groups (*APC, TP53, TTN, KRAS, SYNE1, PIK3CA, MUC16, FAT4, RYR2*, and *DNAH5*). **(F)** The waterfall plot of PRG mutation frequencies in high- and low-risk groups. GO, Gene Ontology; GSEA, gene set enrichment analysis; KEGG, Kyoto Encyclopedia of Genes and Genomes; PRG, pyroptosis-related gene.

The lncRNAs might play an important role in biological processes such as cell differentiation, development, tumor growth, and metastasis of CRC (29, 30). We performed Gene Ontology (GO) and Kyoto Encyclopedia of Genes and Genomes (KEGG) pathway analyses based on differential gene expression profiles in the low- and high-risk groups to uncover relevant functional pathways (differentially expressed genes (DEGs) [|log2 (fold change) | > 0.5 and p< 0.01). The DEGs were enriched in a variety of cellular and biological functions, including external encapsulating structure organization, immunoglobulin complex formation, antigen binding, metalloendopeptidase activity, endopeptidase activity, protein digestion and absorption, TGF-beta signaling pathway, and proteoglycans in cancer (**Figures 9A, B**). The GSEA discovered mainly enrichment of tumor invasion and progression pathways in high-risk groups (**Figure 9C**; **Supplementary Table S6**). Then, changes in the distribution of somatic mutations were explored in the low- and high-risk groups. We found differences in the frequency of mutations in certain genes between the individual groups (**Supplementary Table S7**). The mutation frequencies of *APC, TP53, TTN, PIK3CA, MUC16*, and *OBSCN* were higher in the high-risk group than in the low-risk group (**Figures 9D, E**). However, there were no differences in pyroptosis-related genes between the high- and low-risk groups (**Supplementary Table S7**), and the mutations of pyroptosis-related genes are shown in **Figure 9F**.

### Immune landscape analysis between two pyroptosis-related lncRNA groups

**Figure 10:**
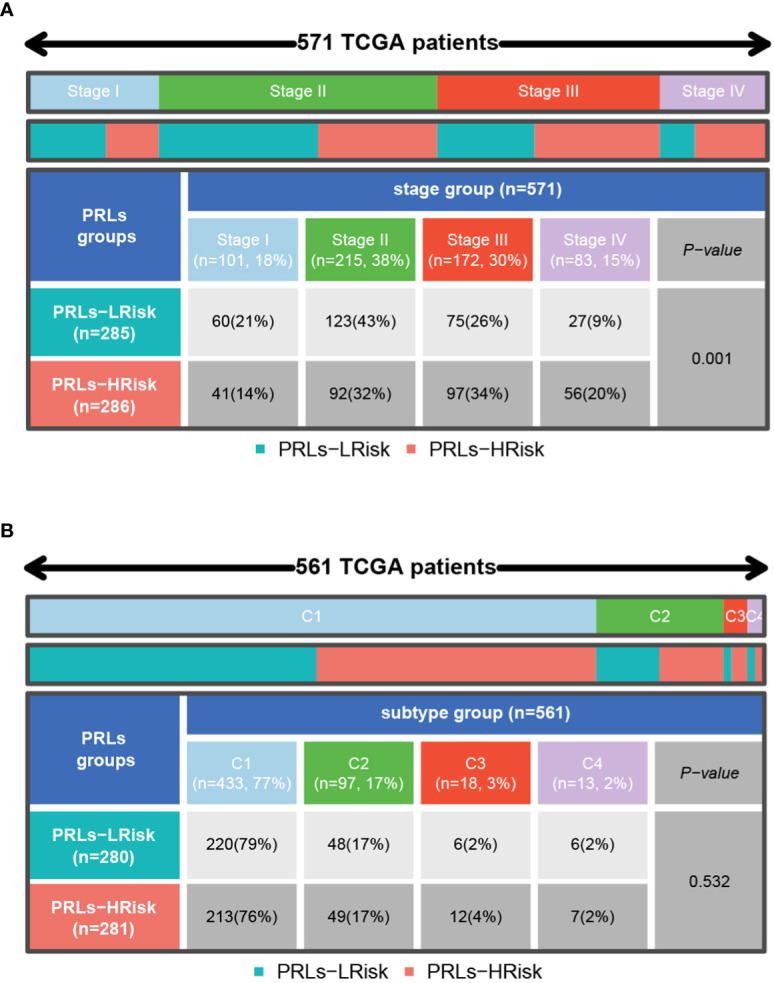
Distribution of stage and pan-cancer subtypes in low- and high-risk score subgroups. **(A)** The proportion of patients with different clinical stages between low- and high-risk groups shown in heatmap and tables. **(B)** The proportion of patients with different pan-cancer immune subtypes (C1, C2, C3, C4, C5, and C6) between low- and high-risk groups shown in heatmap and tables. C1, wound healing; C2, IFN-γ dominant; C3, inflammatory; C4, lymphocyte depleted; C5, immunologically quiet; C6, TGF-β dominant. The comparison analysis between the two risk groups through the chi-square test.

**Figure 11:**
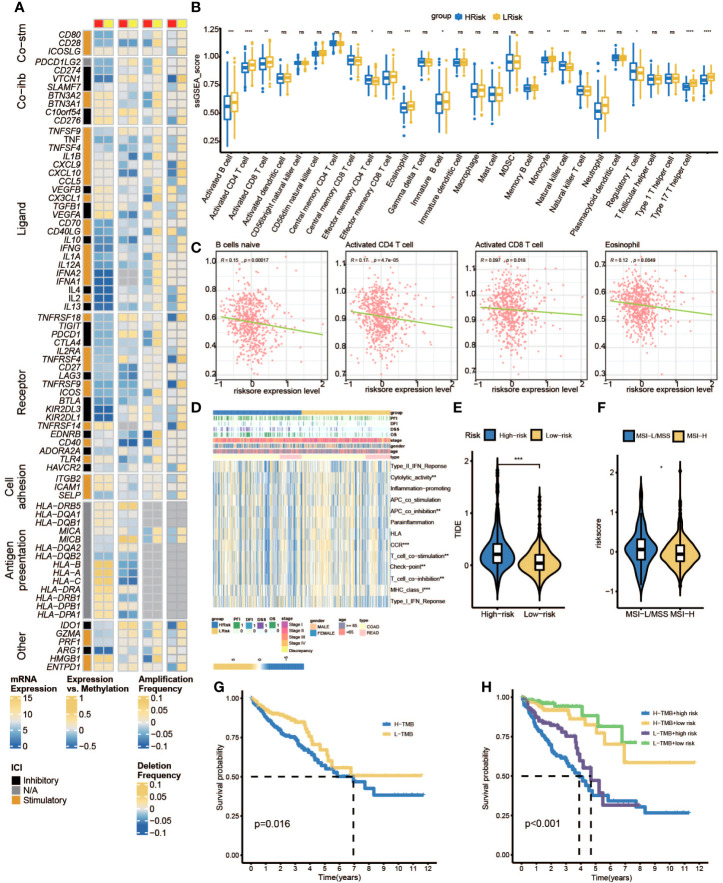
Immune landscape shaped by PRLs. **(A)** Differences in expression of immunomodulators among two pyroptosis-related lncRNA subtypes. From left to right: lncRNA expression; lncRNA expression versus methylation; amplification frequency and deletion frequency for 75 IM genes stratified by PRL subtypes. **(B)** The ssGSEA score group is stratified by high- and low-risk scores. **(C)** The associations between naïve B cell-activated CD4+ T cells, activated CD8+ T cells, eosinophils, and risk scores. **(D)** Heatmap of the associations between clinicopathological features, immune-related pathways, and risk scores. **(E)** Different TIDE scores between high- and low-risk groups. **(F)** Different MSI statuses between high- and low-risk groups. **(G)** K-M curves of patients in high- and low-TMB subgroups. **(H)** K-M curve for H-TMB with high-risk score group, H-TMB with low-risk score group, L-TMB with high-risk score group, and L-TMB with low-risk score group. IMs, immunomodulators; ssGSEA, single sample gene set enrichment analysis; TIDE, Tumor Immune Dysfunction and Exclusion; MSI, microsatellite instability; TMB, tumor mutational burden. * represents p < 0.05, ** represents p < 0.01, *** represents p < 0.001, ns represents no significant.

Firstly, we can observe from **Figure 10A** that there was more proportion of stage III and stage IV patients in the high-risk group, while there were more stage I and stage II patients in the low-risk group (p< 0.001, chi-square test). To characterize the immune characteristics between the low- and high-risk groups, several immune-related analytical methods were performed. Primarily, 565 samples in TCGA cohort were sorted into six subtypes according to pan-cancer immune phenotype set by a previous study (C1, wound healing; C2, IFN-γ dominant; C3, inflammatory; C4, lymphocyte depleted; C5, immunologically quiet; C6, TGF-β dominant) (31). There was no significant difference in the distribution of immune subtypes between the low- and high-risk groups (**Figure 10B**). Generally, immunomodulators (IMs) are critical for cancer immunotherapy, which can unleash antitumor immunity. Several immunomodulator agonists and antagonists have been applied in clinical practice (32). In our study, 78 IMs were included to examine differences in expression levels across PRL subtypes (31, 33, 34). To explore the relationship between gene expression and DNA methylation, amplification, or deletion, Spearman’s correlation analysis was conducted in each PRL subtype. The potential effects of PRL expression levels on TME were tested. Interestingly, gene repression of IMs largely segregated CRCs by PRL groups (**Figure 11A**). Tumor-infiltrating lymphocytes (TILs) have been recognized as a positive prognostic factor in CRC (35). We compared TILs between the low- and high-risk groups to investigate if PRLs could affect immunogenicity and immune infiltration. The results of seven immunocyte-associated algorithms (CIBERSORT, EPIC, Estimate, MCP_counter, Quanti-seq, TIMER, and xCell) are shown in **Supplementary Figure S2**. Notably, activated B cells, activated CD4 T cells, activated CD8 T cells, eosinophils, and type 1 T helper cells were decreased significantly in CRC patients in the high-risk group (**Figure 11B**). A higher risk score negatively correlated with the number of naïve B cells, activated CD4 T cells, activated CD8 T cells, and eosinophils (**Figure 11C**). Furthermore, the expression levels of 13 immune-related pathways in the high- and low-risk groups of CRC patients related to clinical features are listed in **Figure 11D**. Then, TIDE analysis was used to predict the efficacy of immune checkpoint blockade (ICB) therapy. The high-risk group had a higher score of T-cell dysfunction (**Figure 11E**). In addition, deficient mismatch repair (dMMR)/microsatellite instability-high (MSI-H) is of great significance for the diagnosis, prognosis, and treatment of various tumors. CRC patients with MSI-H tumors could especially benefit from immunotherapy (36, 37). In colorectal cancer, which is characterized by microsatellite instability, increased TMB was observed (38). As shown in **Figure 11F**, the patients with MSI-H had a lower risk score. In CRC characterized by MSI, increased TMB was observed. Tumors with highly TMB are more easily recognized by our immune system; current evidence suggests that TMB is associated with the efficacy of ICB therapy, as shown in **Figure 11G**, and patients with low TMB have a higher survival probability. Then, a joint survival analysis of the PRL signature combined with TMB was performed. CRC patients had a higher survival probability in the low-risk group with a lower TMB in TCGA cohort than in the high-risk group with a higher TMB (**Figure 11H**) (39). These results indicated that the eight-lncRNA signature had the potential to predict tumor response to ICB therapy in CRC.

### Estimation of the drug responses with pyroptosis-related lncRNAs in clinical samples

**Figure 12:**
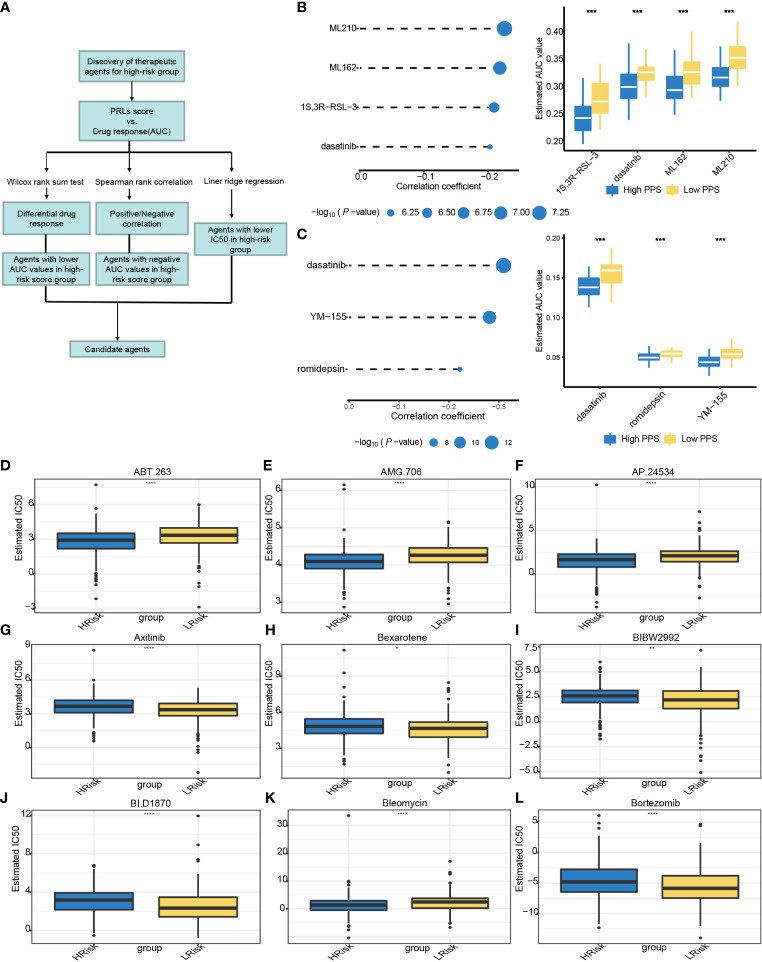
Relationships between risk score and chemotherapeutic sensitivity. **(A)** The outline of the research strategy to explore tumor sensitivity to chemotherapeutic drugs. **(B)** Four CTRP-derived compounds: correlation coefficient of Spearman’s correlation analysis and differential drug response between high- and low-risk score groups. **(C)** Three PRISM-derived compounds: correlation coefficient of Spearman’s correlation analysis and differential drug response between high- and low-risk score group. **(D–L)** The PRLs predict chemosensitivity to chemotherapeutics. CTRP, Cancer Therapeutics Response Portal; PRISM, PRISM Repurposing dataset; IC50, half maximal inhibitory concentration; PRLs, pyroptosis-related lncRNAs. *** represents p < 0.001, **** represents p < 0.0001.

To further explore if PRLs could influence the effect of chemotherapy, we use three different methods to identify candidate potential therapeutic agents in patients with high PRL scores by using the CTRP, PRISM, and GDSC cell line data. The workflow of drug responses in this study is shown in **Figure 12A**. Firstly, the CTRP and PRISM were used to analyze differential drug responses between the high- and low-risk score groups. The correlation of AUC and PRL scores was calculated by Spearman’s correlation coefficient. A negative association was selected because a higher AUC value indicated decreased sensitivity to treatment. The results yielded four CTRP-produced compounds (ML210, ML162, 1S,3R-RSL-3, and dasatinib) and three PRISM-produced compounds (dasatinib, YM-155, and romidepsin). These compounds had a negative association with PRLs and have a lower AUC value (**Figures 12B,C**). Then, based on GDSC, a low-risk score correlated with a higher IC50 value of ABT.263, AMG.706, AP.24534, or bleomycin. The IC50 values of chemotherapeutics such as axitinib, bexarotene, BIBW2992, BI.D1870, and bortezomib were significantly lower in the low-risk score group (**Figures 12D–L**). The eight lncRNAs are expected to predict response to immunotherapy in CRC.

## Discussion

The incidence of CRC keeps rising globally. Because of its malignant characteristics such as frequent relapse, early metastasis, and resistance to radiotherapy and chemotherapy, the prognosis of patients with advanced tumors remains dismal. Furthermore, CRC has high heterogeneity (interpatient, intertumoral, and intratumoral differences), so clinical outcomes among cancer patients are greatly varied. The predictive power of existing molecular markers can be very limited. Therefore, it is of great significance to explore new prognostic molecular markers with great potential. Pyroptosis is a form of non-apoptotic cell death, which has been indicated to be a double-edged sword for innate immunity and antitumor effects (40, 41). For example, pyroptosis-related gene signatures can be effectively applied to the prognosis of CRC patients (19). Notably, lncRNAs are related to tumor progression (42). It is important to explore the potential molecular targets of CRC in-depth. In this study, some of the already identified PRLs involved in the model constructed are already being proven to play a vital role in cancer development. For example, *CCDC144NL-AS1* has been reported to promote the progression of CRC, hepatocellular carcinoma, and osteosarcoma (43–45); lncRNA *MNX1-AS1* is an oncogenic propellant in a variety of tumors, including esophageal squamous cell carcinoma (46); *NKILA* can promote tumor immune escape by interacting with NF-κB in breast cancer (47); *TEX41* promotes the malignant behaviors of skin cutaneous melanoma and lymphoblastic leukemia (48, 49). In addition, recent evidence has validated that *FENDRR* is abnormally expressed in a variety of cancers and associated with advanced-stage CRC and lung cancer (50, 51). *LINC02381*, downregulated in CRC tissues, when its functions were epigenetically silenced, will be an inhibitor of proliferation and viability in colorectal cancer cells (52). However, among these lncRNAs, the function of *Z99289.2* and *LINC02798* is not clear enough.

To investigate the potential biological function of this signature, GO and KEGG pathway analyses were carried out on those identified differentially expressed genes. Interestingly, the eight-lncRNA signature can be primarily enriched in encapsulating structure organization, immunoglobulin complex formation, antigen binding, endopeptidase activity, protein digestion and absorption, TGF-beta signaling pathway, and proteoglycans in cancer.

The TME plays an important role in tumor development, progression, and drug resistance (53). Cancer cells eventually acquire the ability to suppress tumor antagonism of immune cells and evade immune surveillance, leading to tumor progression (37, 54). An immunosuppressive TME is key to promoting tumor development and progression in CRC. Despite the pivotal advance in immunotherapy, the prognosis of CRC remains highly heterogeneous (55–57). The low-risk score group has higher infiltration of activated B cells, activated CD4+ T cells, activated CD8+ T cells, and natural killer cells and thus a better clinical outcome. Thus, PRLs play a positive role in CRC progression and may be a potential molecular biomarker for predicting the efficacy of immunotherapy. Immune checkpoint inhibition therapy can provide rapid and long-lasting treatment for cancer patients, especially those with advanced metastatic diseases, compared with conventional therapies. The TIDE score could become a molecular biomarker to predict response to ICB therapy. In our study, the high-risk group has a higher TIDE score, which reinforces our hypothesis. Mismatch repair defect is present in approximately 15% of all CRC patients, while patients with MSI have upregulation of immune checkpoint proteins and improved prognosis (58). Thus, patients with MSI-H have a lower risk score and might be more inclined to respond to immune checkpoint blockade.

Finally, a diagnostic nomogram model has been constructed with age, tumor stage, T, N, M, and risk score, which improves the ability of PRLs and can be used to identify CRC patients with poor prognoses. In our study, prognostic signature including eight lncRNAs might serve as an independent predictor for clinical outcomes of CRC. It is worth noting that the performance of this signature is better than that of common clinicopathological characteristics in predicting OS of CRC patients, such as age and TNM. Our prognostic model has exhibited satisfactory estimated performance; a patient who has a higher risk score has a worse prognosis, and this was proved in the validation cohorts. In the validation cohorts, the average value of AUC exceeds 0.7. In addition to this, we performed expression and prognosis analyses for each PRL included in the prognostic model. By the result of immunity and drug sensitivity analysis, we anticipate that this signature will help to provide a better understanding of molecular mechanisms underlying CRC pathogenesis as well as shed light on new ideas of targeted therapy for CRC treatment.

In summary, a newly pyroptosis-related long non-coding RNA prognostic model has been constructed, which may provide a better treatment strategy and clinical management for CRC. However, some limitations need to be considered. Firstly, only bioinformatic methods were used to conclude, molecular mechanisms were not explored, and experimental verification was not carried out. Secondly, the sample size should be expanded to observe the differential expression of these lncRNAs between normal and tumor tissues. In addition, the effectiveness of this model in clinical practice is unclear. Thus, we intend to investigate its application in the near future

## Conclusion

To sum up, a novel risk model has been constructed based on eight PRLs (*TEX41, LINC02798, CCDC144NL-AS1, LINC02381, MNX1-AS1, FENDRR, NKILA*, and *Z99289.2*). This signature holds predictive value in CRC patients. A self-developed risk model may provide a new strategy for exploring the pathogenesis of CRC. The eight lncRNAs are expected to predict response to immunotherapy in CRC.

## Data availability statement

The original contributions presented in the study are included in the article/**Supplementary Material**. Further inquiries can be directed to the corresponding authors.

## Author contributions

XC and XL conceived the idea of the manuscript. XC analyzed most of the data. XL drafted and revised the manuscript. KW, YL, MH, HL, XD, and LD supervised the study and revised the manuscript. All authors contributed to the article and approved the submitted version.

## Funding

This study was supported by the National Natural Science Foundation of China (81772557 and 82170525) and the Beijing Municipal Natural Science Foundation (7192087).

## Conflict of interest

The authors declare that the research was conducted in the absence of any commercial or financial relationships that could be construed as a potential conflict of interest.

## Publisher’s note

All claims expressed in this article are solely those of the authors and do not necessarily represent those of their affiliated organizations, or those of the publisher, the editors and the reviewers. Any product that may be evaluated in this article, or claim that may be made by its manufacturer, is not guaranteed or endorsed by the publisher.

## Supplementary material

Supplementary Figure 1 Schoenfeld test was performed to examine the quality of factors that be used to build nomogram. **(A–D)** Schoenfeld individual test for age, N, M and riskscore. The P value is not significant, indicating that the original hypothesis is met.

Supplementary Figure 2 Heat map of immune cell infiltration in low- and high-riskscore subgroup. (Analysis method: CIBERSORT, EPIC, Estimate, MCP-counter, Quanti-seq, TIMER, xCell).

Supplementary Table 1 Identified 33 pyroptosis genes from prior reviews.

Supplementary Table 2 Differently expressed PRLs between normal and tumor sample in TCGA cohort.

Supplementary Table 3 PRLs identified by Spearman analysis.

Supplementary Table 4 Univariate Cox proportional risk regression to filter PRLs that correlated with OS.

Supplementary Table 5 Baseline data sheet for patients in the TCGA cohort.

Supplementary Table 6 GSEA result.

Supplementary Table 7 Differentially mutated genes in the high - and low-risk groups.

## Abbreviations

lncRNAs, long non-coding RNAs; PRL, pyroptosis-related long non-coding RNA; DEL, differentially expressed lncRNA; CRC, colorectal cancer; COAD, colon adenocarcinoma; TCGA, The Cancer Genome Atlas; GEO, Gene Expression Omnibus; AUCs, areas under the receiver operating characteristic curve; OS, overall survival; TMB, tumor mutational burden; TME, tumor immune microenvironment; FPKM, fragments per kilobase of transcript per million mapped reads; GO, Gene Ontology; KEGG, Kyoto Encyclopedia of Genes and Genomes; GSEA, gene set enrichment analysis; CTRP, Cancer Therapeutics Response Portal; PRISM, PRISM Repurposing dataset; IC50, half maximal inhibitory concentration; K-M, Kaplan–Meier.

## References

[1] Siegel RL Miller KD Goding Sauer A Fedewa SA Butterly LF Anderson JC. Colorectal cancer statistics, 2020. CA Cancer J Clin (2020) 70(3):145–64. doi: 10.3322/caac.21601 32133645

[2] Sung H Ferlay J Siegel RL Laversanne M Soerjomataram I Jemal A. Global cancer statistics 2020: GLOBOCAN estimates of incidence and mortality worldwide for 36 cancers in 185 countries. CA Cancer J Clin (2021) 71(3):209–49. doi: 10.3322/caac.21660 33538338

[3] Roerink SF Sasaki N Lee-Six H Young MD Alexandrov LB Behjati S. Intra-tumour diversification in colorectal cancer at the single-cell level. Nature (2018) 556(7702):457–62. doi: 10.1038/s41586-018-0024-3 29643510

[4] He D Zheng J Hu J Chen J Wei X. Long non-coding RNAs and pyroptosis. Clin Chim Acta (2020) 504:201–8. doi: 10.1016/j.cca.2019.11.035 31794769

[5] Fang Y Tian S Pan Y Li W Wang Q Tang Y. Pyroptosis: A new frontier in cancer. BioMed Pharmacother (2020) 121:109595. doi: 10.1016/j.biopha.2019.109595 31710896

[6] Jorgensen I Miao EA. Pyroptotic cell death defends against intracellular pathogens. Immunol Rev (2015) 265(1):130–42. doi: 10.1111/imr.12287 PMC4400865 25879289

[7] Bergsbaken T Cookson BT. Macrophage activation redirects yersinia-infected host cell death from apoptosis to caspase-1-dependent pyroptosis. PLoS Pathogens (2007) 3(11):1570–82. doi: 10.1371/journal.ppat.0030161 PMC2048529 17983266

[8] Mariathasan S Weiss DS Dixit VM Monack DM. Innate immunity against francisella tularensis is dependent on the ASC/caspase-1 axis. J Exp Med (2005) 202(8):1043–9. doi: 10.1084/jem.20050977 PMC2213215 16230474

[9] Burdette BE Esparza AN Zhu H Wang S. Gasdermin d in pyroptosis. Acta Pharm Sin B (2021) 11(9):2768–82. doi: 10.1016/j.apsb.2021.02.006 PMC8463274 34589396

[10] Miguchi M Hinoi T Shimomura M Adachi T Saito Y Niitsu H. Gasdermin c is upregulated by inactivation of transforming growth factor beta receptor type II in the presence of mutated apc, promoting colorectal cancer proliferation. PLoS One (2016) 11(11):e0166422. doi: 10.1371/journal.pone.0166422 27835699 PMC5105946

[11] Beermann J Piccoli MT Viereck J Thum T. Non-coding RNAs in development and disease: Background, mechanisms, and therapeutic approaches. Physiol Rev (2016) 96(4):1297–325. doi: 10.1152/physrev.00041.2015 27535639

[12] Shi X Sun M Liu H Yao Y Song Y. Long non-coding RNAs: A new frontier in the study of human diseases. Cancer Lett (2013) 339(2):159–66. doi: 10.1016/j.canlet.2013.06.013 23791884

[13] Kogo R Shimamura T Mimori K Kawahara K Imoto S Sudo T. Long noncoding RNA *HOTAIR* regulates polycomb-dependent chromatin modification and is associated with poor prognosis in colorectal cancers. Cancer Res (2011) 71(20):6320–6. doi: 10.1158/0008-5472.CAN-11-1021 21862635

[14] Zhang Y Huang W Yuan Y Li J Wu J Yu J. Long non-coding RNA *H19* promotes colorectal cancer metastasis *via* binding to hnRNPA2B1. J Exp Clin Cancer Res (2020) 39(1):141. doi: 10.1186/s13046-020-01619-6 32698890 PMC7412843

[15] Uthman YA Ibrahim KG Abubakar B Bello MB Malami I Imam MU. MALAT1: A promising therapeutic target for the treatment of metastatic colorectal cancer. Biochem Pharmacol (2021) 190:114657. doi: 10.1016/j.bcp.2021.114657 34144008

[16] Silva-Fisher JM Dang HX White NM Strand MS Krasnick BA Rozycki EB. Long non-coding RNA *RAMS11* promotes metastatic colorectal cancer progression. Nat Commun (2020) 11(1):2156. doi: 10.1038/s41467-020-15547-8 32358485 PMC7195452

[17] Ghandi M Huang FW Jane-Valbuena J Kryukov GV Lo CC McDonald ER III. Next-generation characterization of the cancer cell line encyclopedia. Nature. (2019) 569(7757):503–8. doi: 10.1038/s41586-019-1186-3 PMC6697103 31068700

[18] Latz E Xiao TS Stutz A. Activation and regulation of the inflammasomes. Nat Rev Immunol (2013) 13(6):397–411. doi: 10.1038/nri3452 23702978 PMC3807999

[19] Ye Y Dai Q Qi H. A novel defined pyroptosis-related gene signature for predicting the prognosis of ovarian cancer. Cell Death Discovery (2021) 7(1):71. doi: 10.1038/s41420-021-00451-x 33828074 PMC8026591

[20] Karki R Kanneganti TD. Diverging inflammasome signals in tumorigenesis and potential targeting. Nat Rev Cancer (2019) 19(4):197–214. doi: 10.1038/s41568-019-0123-y 30842595 PMC6953422

[21] Man SM Kanneganti TD. Regulation of inflammasome activation. Immunol Rev (2015) 265(1):6–21. doi: 10.1111/imr.12296 25879280 PMC4400844

[22] Wallach D Kang TB Dillon CP Green DR. Programmed necrosis in inflammation: Toward identification of the effector molecules. Science (2016) 352(6281):aaf2154. doi: 10.1126/science.aaf2154 27034377

[23] Subramanian A Tamayo P Mootha VK Mukherjee S Ebert BL Gillette MA. Gene set enrichment analysis: A knowledge-based approach for interpreting genome-wide expression profiles. Proc Natl Acad Sci USA (2005) 102(43):15545–50. doi: 10.1073/pnas.0506580102 PMC1239896 16199517

[24] Geeleher P Cox NJ Huang RS. Clinical drug response can be predicted using baseline gene expression levels and *in vitro* drug sensitivity in cell lines. Genome Biol (2014) 15(3):R47. doi: 10.1186/gb-2014-15-3-r47 24580837 PMC4054092

[25] Lu XF Jiang LY Mang LY Zhu Y Hu WJ Wang JS. Immune signature-based subtypes of cervical squamous cell carcinoma tightly associated with human papillomavirus type 16 expression, molecular features, and clinical outcome. Neoplasia (2019) 21(6):591–601. doi: 10.1016/j.neo.2019.04.003 31055200 PMC6658934

[26] Becht E Giraldo NA Lacroix L Buttard B Elarouci N Petitprez F. Erratum to: Estimating the population abundance of tissue-infiltrating immune and stromal cell populations using gene expression. Genome Biol (2016) 17(1):249. doi: 10.1186/s13059-016-1113-y 27908289 PMC5134277

[27] Newman AM Steen CB Liu CL Gentles AJ Chaudhuri AA Scherer F. Determining cell type abundance and expression from bulk tissues with digital cytometry. Nat Biotechnol (2019) 37(7):773–82. doi: 10.1038/s41587-019-0114-2 PMC6610714 31061481

[28] Jiang P Gu S Pan D Fu J Sahu A Hu X. Signatures of T cell dysfunction and exclusion predict cancer immunotherapy response. Nat Med (2018) 24(10):1550–8. doi: 10.1038/s41591-018-0136-1 PMC6487502 30127393

[29] Kitagawa M Kitagawa K Kotake Y Niida H Ohhata T. Cell cycle regulation by long non-coding RNAs. Cell Mol Life Sci (2013) 70(24):4785–94. doi: 10.1007/s00018-013-1423-0 PMC3830198 23880895

[30] Ye LC Zhu X Qiu JJ Xu J Wei Y. Involvement of long non-coding RNA in colorectal cancer: From benchtop to bedside (Review). Oncol Lett (2015) 9(3):1039–45. doi: 10.3892/ol.2015.2846 PMC4315074 25663854

[31] Thorsson V Gibbs DL Brown SD Wolf D Bortone DS Yang THO. The immune landscape of cancer. Immunity (2018) 48(4):812–+. doi: 10.1016/j.immuni.2019.08.004 PMC5982584 29628290

[32] Tang J Shalabi A Hubbard-Lucey VM. Comprehensive analysis of the clinical immuno-oncology landscape. Ann Oncol (2018) 29(1):84–91. doi: 10.1093/annonc/mdx755 29228097

[33] Yang J Hu LQ. Immunomodulators targeting the PD-1/PD-L1 protein-protein interaction: From antibodies to small molecules. Medicinal Res Rev (2019) 39(1):265–301. doi: 10.1002/med.21530 30215856

[34] Steel JC Waldmann TA Morris JC. Interleukin-15 biology and its therapeutic implications in cancer. Trends Pharmacol Sci (2012) 33(1):35–41. doi: 10.1016/j.tips.2011.09.004 22032984 PMC3327885

[35] Paijens ST Vledder A de Bruyn M Nijman HW. Tumor-infiltrating lymphocytes in the immunotherapy era. Cell Mol Immunol (2021) 18(4):842–59. doi: 10.1038/s41423-020-00565-9 PMC8115290 33139907

[36] Rooney MS Shukla SA Wu CJ Getz G Hacohen N. Molecular and genetic properties of tumors associated with local immune cytolytic activity. Cell (2015) 160(1-2):48–61. doi: 10.1016/j.cell.2014.12.033 25594174 PMC4856474

[37] Chalmers ZR Connelly CF Fabrizio D Gay L Ali SM Ennis R. Analysis of 100,000 human cancer genomes reveals the landscape of tumor mutational burden. Genome Med (2017) 9(1):34. doi: 10.1186/s13073-017-0424-2 28420421 PMC5395719

[38] Schrock AB Ouyang C Sandhu J Sokol E Jin D Ross JS. Tumor mutational burden is predictive of response to immune checkpoint inhibitors in MSI-high metastatic colorectal cancer. Ann Oncol (2019) 30(7):1096–103. doi: 10.1093/annonc/mdz134 31038663

[39] Fancello L Gandini S Pelicci PG Mazzarella L. Tumor mutational burden quantification from targeted gene panels: major advancements and challenges. J Immunother Cancer (2019) 7(1):183. doi: 10.1186/s40425-019-0647-4 31307554 PMC6631597

[40] Tsuchiya K. Switching from apoptosis to pyroptosis: Gasdermin-elicited inflammation and antitumor immunity. Int J Mol Sci (2021) 22(1):426. doi: 10.3390/ijms22010426 33406603 PMC7794676

[41] Wang Q Wang Y Ding J Wang C Zhou X Gao W. A bioorthogonal system reveals antitumour immune function of pyroptosis. Nature (2020) 579(7799):421. doi: 10.1038/s41586-020-2079-1 32188939

[42] Huarte M. The emerging role of lncRNAs in cancer. Nat Med (2015) 21(11):1253–61. doi: 10.1038/nm.3981 26540387

[43] Zhang Y Zhang H Wu S. LncRNA-*CCDC144NL-AS1* promotes the development of hepatocellular carcinoma by inducing WDR5 expression *via* sponging miR-940. J Hepatocellular Carcinoma (2021) 8:333–48. doi: 10.2147/JHC.S306484 PMC8104990 33977095

[44] Wang Y Guo B Xiao Z Lin H Zhang X Song Y. Long noncoding RNA *CCDC144NL-AS1* knockdown induces naïve-like state conversion of human pluripotent stem cells. Stem Cell Res Ther (2019) 10(1):220. doi: 10.1186/s13287-019-1323-9 31358062 PMC6664583

[45] He J Guan J Liao S Wu Z Liu B Mo H. Long noncoding RNA *CCDC144NL-AS1* promotes the oncogenicity of osteosarcoma by acting as a molecular sponge for microRNA-490-3p and thereby increasing HMGA2 expression [Expression of concern]. Onco Targets Ther (2021) 14:5057–8. doi: 10.2147/OTT.S344575 PMC8543027 34707370

[46] Shen Y Lv M Fang Y Lu J Wu Y. LncRNA *MNX1-AS1* promotes ovarian cancer process *via* targeting the miR-744-5p/SOX12 axis. J Ovarian Res (2021) 14(1):161. doi: 10.1186/s13048-021-00910-0 34789303 PMC8596928

[47] Huang D Chen J Yang L Ouyang Q Li J Lao L. *NKILA* lncRNA promotes tumor immune evasion by sensitizing T cells to activation-induced cell death. Nat Immunol (2018) 19(10):1112–25. doi: 10.1038/s41590-018-0207-y 30224822

[48] Chen Z-y Huang J-q Zhu Y Chen Y-s Yu X-f Wang F. Comprehensive analysis of the immune implication of *TEX41* in skin cutaneous melanoma. Dis Markers (2021) 2021:1–11. doi: 10.1155/2021/2409820 PMC8595038 34795805

[49] Orlandella FM Smaldone G Salvatore G Vitagliano L Cianflone A Parasole R. The lncRNA *TEX41* is upregulated in pediatric b-cells acute lymphoblastic leukemia and it is necessary for leukemic cell growth. Biomark Res (2021) 9(1):54. doi: 10.1186/s40364-021-00307-7 34233751 PMC8261931

[50] Zheng Q Zhang Q Yu X He Y Guo W. *FENDRR*: A pivotal, cancer-related, long non-coding RNA. Biomedicine Pharmacotherapy (2021) 137:111390. doi: 10.1016/j.biopha.2021.111390 33761608

[51] Pan H Yu T Sun L Chai W Liu X Yan M. LncRNA *FENDRR*-mediated tumor suppression and tumor-immune microenvironment changes in non-small cell lung cancer. Trans Cancer Res (2020) 9(6):3946–59. doi: 10.21037/tcr-20-2147 PMC8797579 35117761

[52] Jafarzadeh M Soltani BM Soleimani M Hosseinkhani S. Epigenetically silenced *LINC02381* functions as a tumor suppressor by regulating PI3K-akt signaling pathway. Biochimie (2020) 171-172:63–71. doi: 10.1016/j.biochi.2020.02.009 32092325

[53] Hinshaw DC Shevde LA. The tumor microenvironment innately modulates cancer progression. Cancer Res (2019) 79(18):4557–66. doi: 10.1158/0008-5472.CAN-18-3962 PMC6744958 31350295

[54] Rahma OE Hodi FS. The intersection between tumor angiogenesis and immune suppression. Clin Cancer Res (2019) 25(18):5449–57. doi: 10.1158/1078-0432.CCR-18-1543 30944124

[55] Berntsson J Nodin B Eberhard J Micke P Jirström K. Prognostic impact of tumour-infiltrating b cells and plasma cells in colorectal cancer. Int J Cancer (2016) 139(5):1129–39. doi: 10.1002/ijc.30138 27074317

[56] Ijsselsteijn ME Sanz-Pamplona R Hermitte F de Miranda NFCC. Colorectal cancer: A paradigmatic model for cancer immunology and immunotherapy. Mol Aspects Med (2019) 69:123–9. doi: 10.1016/j.mam.2019.05.003 31136750

[57] Mei Z Liu Y Liu C Cui A Liang Z Wang G. Tumour-infiltrating inflammation and prognosis in colorectal cancer: systematic review and meta-analysis. Br J Cancer (2014) 110(6):1595–605. doi: 10.1038/bjc.2014.46 PMC3960618 24504370

[58] Gelsomino F Barbolini M Spallanzani A Pugliese G Cascinu S. The evolving role of microsatellite instability in colorectal cancer: A review. Cancer Treat Rev (2016) 51:19–26. doi: 10.1016/j.ctrv.2016.10.005 27838401

