# Supplementary figures and images for: Pyroptosis-related lncRNAs: A novel prognosis signature of colorectal cancer
Source: Front Oncol. 2022 Nov 30;12:983895. doi: 10.3389/fonc.2022.983895 (PMC9748486; doi:10.3389/fonc.2022.983895)

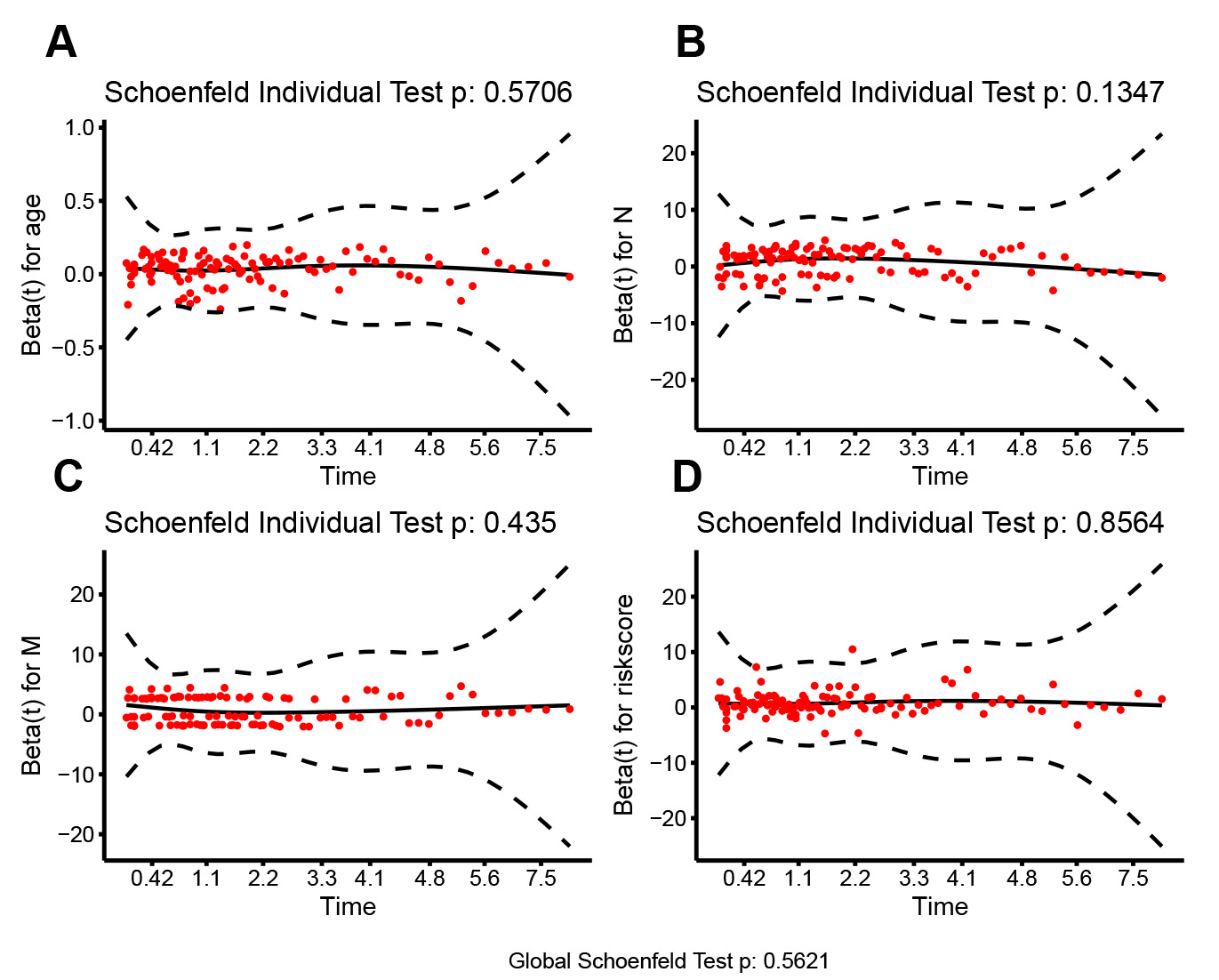

Supplement: Supplementary Figure 1 — Schoenfeld test was performed to examine the quality of factors that be used to build nomogram. (A–D) Schoenfeld individual test for age, N, M and riskscore. The P value is not significant, indicating that the original hypothesis is met. [file Image_1.jpeg]

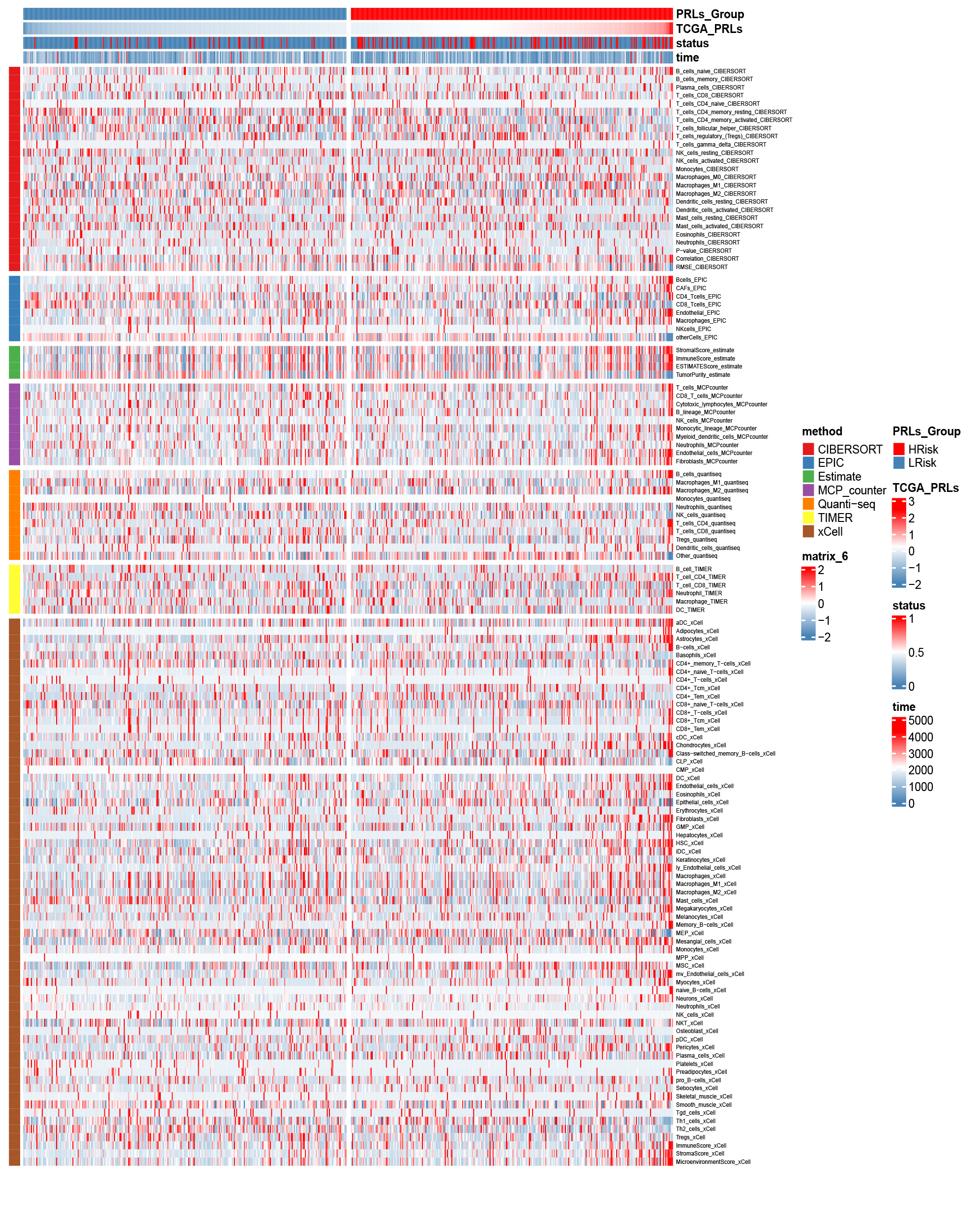

Supplement: Supplementary Figure 2 — Heat map of immune cell infiltration in low- and high-riskscore subgroup. (Analysis method: CIBERSORT, EPIC, Estimate, MCP-counter, Quanti-seq, TIMER, xCell). [file Image_2.jpeg]
