# Supplementary material for: Pyroptosis-related lncRNAs: A novel prognosis signature of colorectal cancer
Source: Front Oncol. 2022 Nov 30;12:983895. doi: 10.3389/fonc.2022.983895 (PMC9748486; doi:10.3389/fonc.2022.983895)
Supplement: Supplementary Table 1 — Identified 33 pyroptosis genes from prior reviews. [file Table_1.docx]

| Identified 33 pyroptosis genes from prior reviews |
| --- |
| AIM2, CASP1, CASP3, CASP4, CASP5, CASP6, CASP8, CASP9, ELANE, GPX4,  GSDMA, GSDMB, GSDMC, GSDMD, GSDME, IL18, IL1B, IL6, NLRC4, NLRP1,  NLRP2, NLRP3, NLRP6, NLRP7, NOD1, NOD2, PJVK, PLCG1, PRKACA, PYCARD,  SCAF11, TIRAP, TNF |

**Supplementary Table 1**: Identified 33 pyroptosis genes from prior reviews
