# Supplementary material for: Pyroptosis-related lncRNAs: A novel prognosis signature of colorectal cancer
Source: Front Oncol. 2022 Nov 30;12:983895. doi: 10.3389/fonc.2022.983895 (PMC9748486; doi:10.3389/fonc.2022.983895)
Supplement: Supplementary Table 2 — Differently expressed PRLs between normal and tumor sample in TCGA cohort. [file Table_2.docx]

|  | Overall | Alive | Dead | p |
| --- | --- | --- | --- | --- |
| N | 571 | 457 | 114 |  |
| **OS** = 1 (%) | 114 (20.0) | 0 (0.0) | 114 (100.0) | <0.001 |
| **OS.time** (median [IQR]) | 1.84 [1.08, 3.00] | 2.00 [1.17, 3.00] | 1.25 [0.60, 3.06] | 0.001 |
| **Age**(median[IQR]) | 68.00 [58.00, 75.50] | 66.00 [57.00, 74.00] | 72.50 [64.00, 78.00] | <0.001 |
| **Gender** = FEMALE (%) | 307 (53.8) | 246 (53.8) | 61 (53.5) | 1 |
| **STAGE**=StageIII_IV (%) | 255 (44.7) | 246 (53.8) | 77 (67.5) | <0.001 |
| **Riskscore**(median [IQR]) | -0.03 [-0.23, 0.18] | -0.08 [-0.26, 0.15] | 0.13 [-0.03, 0.29] | <0.001 |
| **T** = T3_4 (%) | 454 (79.5) | 349 (76.4) | 105 (92.1) | <0.001 |
| **N** = N1_2 (%) | 246 (43.1) | 173 (37.9) | 73 (64.0) | <0.001 |
| **M** = M1 (%) | 133 (23.5) | 85 (18.8) | 48 (42.5) | <0.001 |
